# Health Sciences cultural safety education in Australia, Canada, New Zealand, and the United States: a literature review

**DOI:** 10.5116/ijme.5bc7.21e2

**Published:** 2018-10-25

**Authors:** Donna Lee Marie Kurtz, Robert Janke, Jeanette Vinek, Taylor Wells, Pete Hutchinson, Amber Froste

**Affiliations:** 1 School of Nursing, Faculty of Health and Social Development, University of British Columbia Okanagan Kelowna, Canada; 2 First Nations, Inuit and Métis Cancer Control, Canadian Partnership Against Cancer; 3 Okanagan Indian Band, Community Services and Development, Vernon, Canada

**Keywords:** Cultural safety education, cultural competence, didactic and experiential curriculum; medical and allied health education, Aboriginal and Indigenous health collaboration

## Abstract

**Objectives:**

To review the research literature on cultural safety education within post-secondary health science programs.

**Methods:**

We conducted health and social science database searches from 1996-2016, using combined keywords: cultural competence or safety; teaching or curriculum; universities, polytechnics or professional programs; and Aboriginal or Indigenous. In dyads, authors selected, and reviewed studies independently followed by discussion and consensus to identify thematic linkages of major findings.

**Results:**

A total of 1583 abstracts and 122 full-text articles were reviewed with 40 selected for final inclusion. Publications from Australia, Canada, New Zealand and the United States described curriculum development and delivery. A variety of evaluation approaches were used including anecdotal reports, focus groups, interviews, course evaluations, reflective journals, pre-post surveys, critical reflective papers, and exam questions. Duration and depth of curricular exposure ranged from one day to integration across a six-year program.  Changes in student knowledge, attitude, self-confidence, and behaviour when working with Indigenous populations were reported. Cultural safety education and application to practice were shown to be linked to improved relationships, healthier outcomes, and increased number of Indigenous people entering health education programs and graduates interested in working in diverse communities.

**Conclusions:**

This review provides a summary of multidisciplinary didactic and experiential instructional approaches to cultural safety education and the impact on students, educators and Indigenous people.  Institutional support, strategic planning and cultural safety curriculum policy within post-secondary settings and community engagement are imperative for positive student experiences, advocacy, and actions toward health equity and improved health for Indigenous people and communities.

## Introduction

Health inequities and disparities and gaps in health provision for Indigenous people exist globally. Australia, Canada, New Zealand, and United States share similar colonizing histories and are leading the way in health science curriculum and application to practice to address these issues. Medical and allied health professional organizations and educational programs in Canada report needed changes in curriculum and practice for improved health of Aboriginal Peoples (First Nations, Metis, Inuit).^1^ Collaboration and commitment to address gaps include full involvement of Aboriginal people in decision-making regarding the health of their peoples. The Truth and Reconciliation Commission of Canada [TRC] recommends cultural competency training for all health-care professionals and calls upon schools to provide skills-based training in intercultural competency, conflict resolution, human rights, and anti-racism.^2^  

Within this paper, terminology and meanings used in referring to the original or First Peoples are important. The Canadian government has categorized the original people of North America by one term Aboriginal, and three distinct groups: First Nations (historically referred to as Indian), Métis and Inuit.^3^ However, Aboriginal is linked to colonialism and destruction of indigenous identities, ownership of land, loss of language, and other colonial acts, and is being used less. The term Indigenous commonly refers to Aboriginal peoples globally,^4^ regardless of borders, Constitutional or legal definitions and is in keeping with Indigenous rights movements.^5^ To respect the terms used by the authors of papers included in this review, the terms Aboriginal or Indigenous, are used in accordance with the term used by cited authors.

Culture, in the context of cultural competence and cultural safety includes, but is not restricted to, age or generation; gender; sexual orientation; occupation and socioeconomic status; ethnic origin or migrant experience; religious or spiritual belief; and disability.^6^ Cultural competence and cultural safety literacy is debated at an international level. Therefore, differentiation between terms and a paradigm shift in the understanding of how healthcare students and providers embody culturally safe healthcare is needed.^7^

Cultural competence is the mastery of a set of measurable skills, knowledge, attitudes and behaviours in which practitioners begin to become self-aware of their own culture in providing quality care to diverse populations.^8^  This awareness, solely determined by the practitioner, enables effective work in cross-cultural situations but does not address the inherent power imbalance between the recipient of the care and the healthcare provider.^9^ Cultural safety extends beyond cultural competence, and focuses on the “social, structural and power inequities that underpin health inequalities/disparities”^10^ and is determined and felt by both service-users and practitioners. 

Cultural safety education, stemming from concerns about the health status of Māori people in New Zealand, prepares practitioners to challenge unequal power relationships that perpetuate health inequalities and disparities of individuals, families and communities.^11^ It fosters sharing of power in that the recipient (patient/community) of healthcare determines how safe they feel during the service encounter.^12^ Culturally safe practice recognizes historical and contemporary colonization, and societal, institutional, and political power structures that continue to undermine Indigenous people’s role in their own care within the healthcare system.^13^  A culturally safe environment is one that acknowledges and respects all aspects of a person’s life and does not lessen or ignore that person’s identity, uniqueness or power as a human being.^7^

For many healthcare disciplines, culturally safe concepts are being included in the education and training of healthcare administrators, providers and educators, to support the development of cultural competencies, shared provider-recipient decision making, and better care that supports healthier outcomes. This includes the adoption of cultural safety within several disciplines including nursing, medicine, occupational and physical therapy, social work, sociology, anthropology, education, pharmacy, and health.  For many of these medical and allied health disciplines, this type of education is  now a requirement for program accreditation and approval to address health gaps between populations.^10^ Educational initiatives are required for healthcare and social service providers to better understand and apply culturally safe principles to their practice, and to reduce healthcare inequities of Indigenous peoples by improving their healthcare experiences^14^^,^^15^ and a review of literature would inform existing and upcoming curricula and programs implementation. 

The terms cultural competence and cultural safety are used according to the terminology found in the articles critiqued.  There is merit in focusing on the cultural competence training from a specific cultural perspective, in this case Aboriginal, including all Indigenous peoples, as it highlights best practices for cultural integration within the curricular development process.  As identified in a cultural competence literature review by Beach and colleagues,^16^ there is significant evidence that curricula teaching specific cultures and worldviews rather than general concepts, may improve care provider knowledge, attitudes, and skills, and patients’ experiences of healthcare delivery.  

While still important, patients’ rating of care should not be confused with healthcare outcomes.  Accordingly, a review by Lie and colleagues, concludes minimal evidence linking cultural competence training to healthcare outcomes, yet suggest there is a “trend in the direction of a positive impact” on patient outcomes.^17^ More recently, Renzaho and colleagues’ review could not empirically support the existence of this trend, finding only two studies that attempted to measure impacts of cultural competent practice on patient outcomes and concluded that to assess the effects properly, more research is needed.^18^

Regarding Indigenous cultural competence training, there were two current literature reviews specifically related to this topic. Downing and colleagues reviewed cultural training of professional health workers in Australia and found most Indigenous cultural training is based on ‘cultural awareness’.^19^ Of the three studies that assessed change, two found positive changes while the only study using a control group, found no effect.  Because this study found poor effectiveness of Indigenous cultural training programmes in Australia, the authors suggest a ‘cultural safety’ based model for education. Indeed, the cultural safety model shifts training away from teaching about culture (ethnicity and/or anthropological) exclusively and examines personal and professional relational power imbalances and identity, offering the potential for improved changes in healthcare practitioner knowledge, attitude, skill, and provision of quality health services for Indigenous Australians.^19^

Ewen and colleagues reviewed interdisciplinary Indigenous health curricula for medical, nursing, dental, and pharmacy students aiming to improve Indigenous health and engagement with health practitioners and health systems.^20^ Unlike Downing and colleagues,^19^ and similar to our review, Ewen and colleagues^20^ focused on instruction at the university-level while Downing and colleagues,^19^ focused on Indigenous health-related curricula in a broader sense, rather than specifically on cultural competence training.  Based on our decade-long and continuing involvement in local, national and international curriculum development, implementation and/or evaluation of cultural safety education. In upcoming publications, we offer what we have learned from students, educators, health care providers and Indigenous teachers, people and patients about how cultural safety education and application to practice. Students report increased empathy, humility, and allyship as a result of self-reflection of their biases and prejudices, recognition of power and privilege related to education and health access, treatment and outcomes, a deeper understanding of generational impacts of historical and contemporary colonialism on the health and wellbeing of Indigenous people globally, and the role they have in advocacy and change. 

The purpose of this review is to improve cultural competency/safety literacy, inform health science students, educators, providers and decision makers about the application of cultural safety when working with Indigenous people. We provide a synthesis of multidisciplinary didactic and experiential cultural safety curriculum approaches in which health science students begin to understand better and become more able to work respectfully with Indigenous people and communities to collectively address health inequities and disparities.  

Our review differentiates findings from Ewen and colleagues^20^ and Downing and colleagues^19^ by including a more comprehensive and recent analysis of data sources beyond those previously searched: PsycINFO (behavioural sciences and mental health), ERIC (educational research), as well as allied health such as social work and social services literature data sources. 

## Methods

Data Sources and Search Strategies: Our literature search was executed in the following seven databases: Medline, Embase, CINAHL, ERIC, PsycINFO, Social Services Abstracts, and Social Work Abstracts.  A combination of free text and controlled vocabulary (subject heading) searching was used and tailored to each database. The search focused on combining the following broad concepts: (1) cultural competence, or safety; (2) teaching or curriculum; (3) universities, polytechnics, or professional programs; and (4) Aboriginal (e.g. 1 AND 2 AND 3 AND 4).  Synonyms for these concepts were also incorporated into the search. These broad concepts were modelled on the literature reviews discussed earlier,^19^^,^^20^ while the synonyms were generated from several sources including author expertise, the wider literature in this area, and terms related to specific areas of geography we wished to include (e.g. the term polytechnic being used heavily in Australia and New Zealand). When available, database limiters were applied to retrieve only articles in English with an abstract available. The results from the database searches were supplemented with articles identified through the screening of bibliographies - in particular the review papers discussed earlier.^19^^,^^20^ Forward citation searching was also conducted on several key articles to ensure the original search was sufficiently exhaustive. A total of 21 additional articles were identified through this process.

### Eligibility Criteria

The following inclusion criteria were used: 1) program discussed or evaluated was geared towards developing culturally competent practitioners who work specifically with Indigenous populations and 2) program was based in a post-secondary institution with either undergraduate or graduate degree healthcare or social services students as the principal participants. Articles were excluded based on the following criteria: 1) not published in English; 2) no abstract available; 3) abstract only available (no full article for review).

### Article Review

In total, 2558 articles were retrieved in the searches using the inclusion/exclusion criteria with 21 identified post-search. All were loaded into RefWorks, a bibliographic management software package.  Duplicate articles were removed leaving 1,583 abstracts to be screened. Teams of researchers and student authors were involved in the review process. Four of the six authors are Indigenous, with at least one Indigenous team member on each team. Initially, two teams consisting of two researchers, individually reviewed abstracts from 1996 – 2011 and one team of two researchers, reviewed abstracts from 2012-2017 individually. Each review was followed by a discussion with the other researchers to resolve differences and to ensure articles met inclusion/exclusion criteria.  As a result, 122 articles were recommended for a full-text review. A standardized extraction table was developed based on tables used in previous literature reviews and discussion among members of this review team. Two authors independently read full articles and extracted to the table to ensure the accuracy of extraction and review process. Indigenous student research assistants, not involved with the initial literature search or development of eligibility criteria or reviews, merged the independent extractions into a common table. The two lead authors then worked together to finalize inclusion, condense extractions and enter into the extraction table. A total of 40 articles met the eligibility criteria summarized in Appendix 1 - Studies Selected for Review. They are categorized by author; publication date; design/method; setting (country); student sample characteristics (field of study); curricular development; curricular delivery; and outcomes/findings. 

## Results

Literature in this area is a more recent phenomenon, with 14 of the 40 articles published in 2014 and 2015 (n=14). The majority (n=19) originated in Australia,^21^^-^^39^ with the remaining from the United States (n=11),^40^^-^^50^ Canada (n=6),^51^^-^^56^ and New Zealand (n=4).^57^^-^^60^ A large proportion of disciplines focused exclusively on students in nursing (n=11), ^24^^, ^^27^^, ^^28^^, ^^34^^, ^^43^^, ^^48^^-^^51^^, ^^59^^, ^^60^ with others in medicine (n=6), ^21^^, ^^25^^, ^^32^^, ^^35^^, ^^38^^, ^^58^ dentistry (n=2), ^31^^, ^^57^ pharmacology (n=2),^45^^, ^^46^ psychology (n=2),^22^^, ^^37^ social work (n=2),^23^^, ^^42^ audiology (n=1),^52^ midwifery (n=1),^39^ and the majority of an interdisciplinary makeup (n=13).^26^^, ^^29^^, ^^30^^, ^^33^^, ^^36^^, ^^40^^, ^^41^^, ^^44^^, ^^47^^, ^^53^^, ^^54^^-^^56^Curriculum delivery methods varied widely, with classroom instruction and practicum experiences most utilized.  Just over half of the articles did not report the involvement of Indigenous people in either curricular development or delivery.  However, more involvement has occurred in more recent years, 2013 to 2016, compared to 2006 to 2013. For example, interaction with students,^56^^, ^^58^ coordinators of local Indigenous community-based activities,^23^^, ^^24^^, ^^26^^, ^^58^ curriculum design,^21^^, ^^25^^, ^^48^^, ^^53^ advisors,^23^^, ^^26^^, ^^34^^, ^^37^^, ^^38^^, ^^40^^, ^^52^^, ^^55^ and as classroom instructors. ^26^^, ^^29^^, ^^30^^, ^^34^^, ^^37^^, ^^49^^, ^^55^^, ^^59^ Although few articles described the use of methodological approaches, such as grounded theory and phenomenology, rigorous evaluation of the effects of the curriculum was not present. Most articles simply described the development and/or delivery of the curriculum without using standardized evaluation methods. A variety of curriculum and course evaluation measures, such as formal and informal, summative, formative, reflective, written and verbal feedback were used. For example, anecdotal reports, focus groups, interviews, course evaluations, reflective journals, pre-post surveys, electronic surveys, critical reflective papers, and exam questions. Duration and depth of curricular exposure ranged from one day to integration across a six-year medical education program.  To provide more detail, the following articles were synthesized qualitatively and grouped per curricular delivery or program approach to help highlight common thematic linkages.

Curriculum Delivery - Didactic and Experiential: Articles reviewed described didactic or experiential practice curricula or programs to prepare healthcare students to become culturally competent or culturally safe when working with Indigenous populations. A common theme suggests as students’ self-knowledge and Indigenous traditional knowledge grew, they became more accepting of traditional healing practices, and their contribution to improved health and well-being.

Walton found that interdisciplinary health science students can learn and offer cultural awareness interventions with limited teaching, however they fail to have a deeper understanding of cultural safety, power differential and the ability to transfer this knowledge from learning to practice.^47^ Several articles reported that cultural awareness only acknowledges difference and does not provide adequate teaching about application to practice. Nor does it teach respectful, equitable distribution of power for both healthcare provider and recipient. For example, in Australia, Chiodo found some students resisted content related to Indigenous culture, inequality, diversity, and relevance to practice.^22^ Hendrick and colleagues used the term ‘educational blind spot’ for students who lacked awareness of historical and cultural impacts on the everyday lives of Indigenous Australian people across their lifespan.^26^ However, several articles reviewed describe significant and relevant learning outcomes for health professional students. Warren found nursing students with cultural safety education, were able to self-analyze and recognize power structures and political/historical contexts for Maori people^.60^ Isaacson reported nursing students who had an immersion experience became significantly more aware of healthcare power imbalances for American Indian people.^43^ Hart and colleagues noticed nursing student knowledge and understanding of Australian Indigenous communities increased their confidence and cultural humility during practicum experiences.^24^ Hunt and colleagues also found improved confidence, as well as decreased negative attitudes after completing a course unit of Australian Indigenous history, culture and health.^27^ Issacs and colleagues’ nursing student survey responses suggested increased knowledge in Indigenous health, however, cultural desire, an intangible concept, was not accurately measured during the study.^28^ Issacs and colleagues suggest a longitudinal study may better assess change in cultural desire over time.

Amundson and colleagues,^40^ Bernhardt and colleagues,^52^ Hendrick and colleagues,^26^ Jarvis-Selinger and colleagues,^55^ Pickrell,^45^ and Smith and colleagues,^38^ describe the importance of university-community partnerships in the co-development of interprofessional courses of classroom and practicum experience. Most found consultation between faculty, students, Indigenous community practitioners and community representatives, critical to successfully meeting learning needs of students, and as mutually beneficial for the community. Hudson and Maar suggest community-level support is needed to strengthen immersion experiences. Aboriginal community members in Canada shared that they found working with students a positive experience and looked forward to ongoing partnerships and student placements.^54^ In addition, maintaining respectful relationships for deeper understandings of them as Aboriginal people and each other was important. Authors stressed that to ensure successful community-based partnership, genuine support by faculty and organizational administration is critical.^54^ Thus, Joyce’s insight of nurse education in New Zealand twenty years ago remains relevant today - educators need to have considerable experience and skill in dealing with student attitudinal issues both in the classroom and practice areas in order to build and maintain cultural safety within healthcare.^59^

Arnold and colleagues reviewed a pilot project in Canada in which the provincial registered nursing Professional Standards in Nursing were used to plan and evaluate a course.^51^ They concluded a cultural safety based didactic and short cultural immersion nursing course, delivered in partnership with local Aboriginal populations, can be a transformative experience for students. Prout and colleagues reported interdisciplinary students in Australia found immersion experiences deepened their learning more than didactic methods.^36^ However, for students to be better prepared for immersion experiences, Hudson and Maar suggested Canadian medical and nursing education curricula needed to include more cultural learning prior to immersion.^54^

Benson and colleagues,^21^ and Mak and colleagues,^32^ evaluated medical student learning in didactic and cultural immersion courses. Students reported deeper understandings of and ability to work with Aboriginal people that they felt would not have occurred without both components and that total immersion in Australian communities was critical. These authors reported graduates of the program were more interested in career choices in rural/remote areas and some successfully recruited in such positions difficult to fill. 

Wittig surveyed American nursing students to determine if didactic course content and practice experiences supported their development of knowledge, attitudes, and skills to provide cultural competent health.^50^ Student responses reflected theoretical understanding of culturally competent care and application of theory to practice.  However, students stated they wanted to have more teaching by an interaction with Native American people. Similarly, Roche and colleagues,^45^ and Roche,^46^ reported on courses for pharmacy students that combined intellectual learning and face-to-face interactions with Native American people.  Personal reflection (journaling about their learning and experiences) was valuable for students and led them to find ways to continue interactions with the population after graduation (i.e. volunteering and employment).^45^ Effective engagement with Indigenous people and positive responses from the community, often attested to respectful attitudes and cultural safety. Morrissey and Ball discussed pharmacy, and clinical science student empathy toward Indigenous people in Australia improved after training and community visits.^33^ Broughton described this outcome in a New Zealand university-community partnership dental program that included culturally appropriate Indigenous content and practicum experiences that were embedded throughout a four-year program.^57^ Lalloo and colleagues stated that dental student online responses and journal reflections reported positive learning experiences in rural Australia yet improvements needed to be done by stakeholders to maintain and enhance experiences.^31^

Nash and colleagues reported on the nursing aspect of an Australian interdisciplinary university-community partnership that involved extensive consultation with Indigenous community staff and health expert members.^34^ Consultation included intensive staff education about the constructs of power and discrimination as they pertain to cultural competence and safety concepts. Ranzijn and colleagues also found success in a university-community partnership cultural competence undergraduate psychology course.^37^ The inclusion of Australian Indigenous people as reference group members, co-teachers and cultural competence trainers for academics teaching the course, was largely beneficial. This lower resistance in student and faculty willingness to learn about Indigenous health, knowledge, and cultural safety can result in a large positive shift in acceptance of Indigenous people.

Community-based Practicums and Cultural Immersion Experiences: In the literature reviewed, community-based practicums and immersion programs were aimed to familiarize students and faculty with cultural competence skill application, interdisciplinary teamwork, common understanding of partnership relationships, and ways to address the shortage of professionals working in underrepresented communities or agencies. However, Pickrell’s report of psychology, occupational health and nursing students’ immersion experience in an American Indian community in the United States, found minimal evidence reporting immersion in one culture enables students to apply learned culturally sensitive knowledge when working with other cultures.^44^ Similarly, Duthie and colleagues found graduate social work students in Australia who attended a one day cultural immersion, were unable to fully grasp the importance of Indigenous community engagement.^23^ However, Cross and colleagues reported designated graduate social work student experiences in American Indian child welfare placements increased their cultural responsiveness in the community.^42^ Given the majority of Indigenous people living in urban areas, when Indigenous health content and practice experiences were embedded in a four-year (medical) program in Australia, Paul and colleagues found challenging stereotypical attitudes can occur without an immersion.^45 ^

The majority of articles clearly described the mutual benefits of cultural immersion for the student, university, and community. Warner stated nurse educators employ a variety of teaching and learning strategies to prepare culturally competent healthcare professionals.^48^ The success of their cultural immersion model expanded community partnerships with local Indigenous healthcare providers seeking out learning activities and ceremonial events in which students could participate. Warner stated this format increased student requests to enroll in the course.  Additionally, Bender and Braziel’s interdisciplinary rural practice framework increased student acknowledgement of differing beliefs and practices.^41^ This approach assisted students in designing healthcare delivery initiatives relevant to American Indian people and prepared them to work in rural areas. Dowell and colleagues found after a one-week cultural immersion program coordinated by local Maori health providers and Elders; students were more able to understand the importance of collaboration with communities in the identification of local community cultural issues and public health needs.^58^ From this experience, more medical students sought employment in Maori rural communities. Kline and colleagues reported interdisciplinary students who attended an Aboriginal community immersion summer camp in Canada led by local community members as instrumental for student learning about communication, university collaboration and the need to integrate Aboriginal perspectives in curricula.^56^

Cultural safety education challenges students to not only learn intellectually but also relationally and emotionally.  Jackson and colleagues reported that content delivered by both Indigenous and non-Indigenous Australian nurses, was perceived by students as transformative, profound, and deeply emotional.^29^ Cultural safety education also increased students’ preparedness to advocate for and communicate with Indigenous people, and students felt their learning transformed them beyond preparedness toward feeling they had a new found personal priority to make changes towards improved Indigenous health.  In some cases, Indigenous communities found engaging with students encouraged their community members to enter healthcare professional education programs and consequently increase the number of Indigenous healthcare professionals working to improve the health and wellness of their communities.  

Hays contended, improved healthcare for specific populations is dependent on successful immersion experiences and partnerships with Indigenous community members.^25^ Australian and Torres Islander community member active involvement in collective decision-making processes such as membership on committees, selection of students and staff, and curriculum design is critical. 

Online Learning: Online or web-based post-secondary curriculum delivery is often the only choice for healthcare students who live in rural or remote areas. Carter and Rukholm described a university-community partnership that developed a web-based course designed to change attitudes regarding interprofessional health education.^53^ Noted changes included an increase in the number of health professionals with interprofessional education and an improvement in collaborative patient-centred care approaches.  Positive student and faculty feedback, Elder requests to volunteer in making additional videos, and plans to deliver the curriculum to undergraduate medical students, demonstrated successful delivery. The authors contended that culturally appropriate principles and practices for curriculum development and delivery cannot be generalized and are more likely to be successful and beneficial when local Aboriginal people are involved.

To make cross-cultural courses more accessible, Wendler and Struthers,^49^ and Kickett and colleagues,^30^ describe adapting courses to a web-based asynchronous course. To enhance experiential learning, Wendler and Struthers, required students to interface with numerous people from diverse populations. Students and faculty indicated they gained deeper insights into the creation of relationships and alliances that would be beneficial in the future. Conversely, although students rated a piloted post-immersion online course highly, Hudson and Maar identified multiple pedagogical difficulties such as student disinterest and lack of meaningful discussion, poor accessibility, and connectivity.^54^ Although online learning has some challenges, it may be the only option for student engagement and learning in some circumstances.

## Discussion

In this critique of cultural competency and cultural safety education literature, we focused on cultural safety didactic delivery and face-to-face experiential learning for undergraduate and graduate medical and allied health and/or social service studies students across four countries. It is apparent that there is a broad range of methods for teaching cultural safety curriculum and each has strengths.  The impetus for cultural safety education and training is to improve the health of Indigenous people, to address the shortage of practitioners educated and prepared to provide culturally safe services in rural and remote Indigenous communities, and to encourage Indigenous people to enter medical and allied health professions.  Findings from this review have implications for cultural safety curriculum developers, teachers and researchers, and community members that have roles in education, practice and health education policy.  This review highlights that literacy and the meaning of cultural safety concepts and terminology is often not widely understood as different from cultural competency, or in relation to populations other than Indigenous.  Although findings support the importance of cultural safety education for student attitude and behaviour change in health sciences, the importance of collaborative partnerships with Indigenous people is key for successful program delivery and sustainability.  Institutional support at all levels of department leadership in providing time, resources, strategic planning and policy within the post-secondary setting is also imperative for successful curriculum delivery, ongoing community engagement, positive student experiences, and increased interest in advocacy, health equity and actions to improve health for Indigenous people and communities.

Implementation science or evidence-based research of the impact of culturally safe practice on improved health outcomes for Indigenous people is minimal and a current debate.^18^ The development of comprehensive tools to measure the experience of culturally safe practice is difficult, in that variables that influence outcomes are dependent on multifaceted contexts. These problematic contexts are: a) involvement of Indigenous healthcare providers and community members in the development; b) delivery and evaluation of cultural safety education; c) maturity of healthcare students; d) previous personal and professional experiences of the student; e) levels of unconscious racist attitudes; f) the unique and diverse populations healthcare professionals encounter; and g) the perpetuation of structural and societal violence within organizations.^16^  

The application of cultural safety concepts and knowledge for non-racist, non-discriminatory healthcare delivery depends on several factors.^9^ Without a continuum of system-wide, culturally safe approaches by medical and allied healthcare professionals and decision and policy makers, culturally safe practice may be viewed as anecdotal, an individual experience, and not evidence-based. Evidence-based research is largely quantitative in nature while qualitative research is widely considered at a lower level of evidence.^61^ However, for those accessing and receiving healthcare, one’s positive culturally safe experience is possibly the most critical measurement of the successful outcome of cultural safety education and practice. Thus, cultural safety education in the context of Aboriginal health can only be defined as culturally safe if it is perceived as such by a specific individual or community.^9^ This clearly identifies that community involvement and perspective is critical, pedagogically and relationally relevant, and directly related to the success of education and training.  This captures how critical it is that the learning environment for this curriculum must be one that is culturally safe; culturally safe for Indigenous teachers, students, and faculty who are involved.  This is a mindful process of navigating and honoring Indigenous and Western perspectives, and what we have learned in our eight years of delivering an experiential cultural safety curriculum, is that learning is not always comfortable, but if the cultural safety curriculum is offered through a lens of cultural safety, the environment can be transformed into one where people feel safe to teach and learn from each other.  

We concur with Ewen and colleagues that evidence over the long-term is needed to demonstrate and evaluate positive patient health outcomes as a result of cultural safety education, while also measuring the impact on the learner.^20^ Cultural safety is an outcome lived through self-reflection, truly listening to each other and sharing respect, meaning, knowledge and experience, experiencing empathy, and ensuring dignity in our everyday relationships. We encourage researchers, communities, institutions, policy makers and educators to begin or continue to engage in knowledge translation to strengthen the evidence for cultural safety that is inclusive of all people. 

### Acknowledgements

We acknowledge partial funding from the University of British Columbia Faculty of Health and Social Development Teaching and Innovation Learning Grant to support student scholarly work.

### Conflict of Interest

The authors declare that they have no conflict of interest.

## Supplementary material

Supplementary file 1 Appendix 1. Studies Selected for Review
