# Supplementary material for: Health Sciences cultural safety education in Australia, Canada, New Zealand, and the United States: a literature review
Source: Int J Med Educ. 2018 Oct 25;9:271–85. doi: 10.5116/ijme.5bc7.21e2 (PMC6387770; doi:10.5116/ijme.5bc7.21e2)
Supplement: Supplementary file 1 — Appendix 1. Studies Selected for Review [file ijme-9-271-S1.pdf]

Appendix 1.

Studies Selected for Review:

Description of 40 studies reporting indigenous-based cultural safety educational initiatives

Author, Year	Design/Method	Student Sample Characteristics	Curricular Development	Curricular Delivery	Outcomes / Findings
Amundson and colleagues (2008) [37]	Evaluation; Pre/post-test questionnaire; Reflective journaling (Glaser's Grounded Theory)	United States; n=46 Medical, PT, OT, Lab Science, Social Work, Nutrition, Radiology, Psychology 10% American Indian; 90% Caucasian	Advisory council (reservation, college, university, Native American Programs, National Resource Center on Native American Aging)	Two sites – 4 week summer internship: American Indian history, social, political, culture, and activities; community projects; engage students in local activities overseen by Aborigional community coordinators	Increased ability to identify cultural differences; communicate with non-English speaking patients; foster interdisciplinary interactions/community relationships; interest in reserve/under-served communities work post-graduation. Program success relies on institutional/faculty support
Arnold and colleagues (2008) [48]	Description with anecdotal evaluation	Canada; n=12, 4th year Nursing; n=12, Community members	Not discussed	Cultural immersion weekend; community project. Campus tour for community learners to meet staff and attend career fairs highlighting nursing as a career choice	Stronger relationships with community; younger community members saw nursing as a career choice; nursing students gained better understanding of Aboriginal health/effects of colonialism
Bender & Braziel (2004) [38]	Description	United States; Five Universities; Medical, Nursing, OT, PT, Physician Assistant, Dental, Optometry, Social Work, Dietetics, Pharmacy	Developed by university/ community/Health Education Centers	One-month clinical mentor /preceptorship; interdisciplinary teams; weekly cultural issues lecture; attitude changes/ expectations paper assignment	Students more aware of impact of culture, and isolation as barriers in rural healthcare delivery
Benson and colleagues (2015) [18]	Interpretative phenomenological analysis; inductive thematic analysis	Australia; Medical students n=23	Developed by university; student-run health groups, with community members/Aboriginal people and Health Board and Aboriginal Community Health Service staff, school, playgroup, community	2-day visit to remote Aboriginal community; participation in activities within clinic and in community	Personal growth; increased comfort to advocate for Aboriginal people and provision of culturally appropriate care; understanding of cultural respect, determinants of health improved
Bernhardt and colleagues (2011) [49]	Evaluation; Focus groups, student/faculty questionnaires; Community interviews	Canada; Audiology, Speech-Language Pathology Science	Advisory group (First Nations academic, Aboriginal/non-Aboriginal community representatives, practitioners, project coordinator, part-time student assistants)	One credit, 8-month course; face-to-face class meetings; student community visits; website for relevant articles, announcements, discussion forum	Students had broader understanding of culture and more prepared to work with Aboriginal populations in future
Broughton (2010) [54]	Description; Evaluation; one final exam question	New Zealand; Dentistry	Curriculum based on Faculty of Dentistry Maori Strategic Framework/Otago and South Island	Integration of content over four years; classroom and workshops; practicum experience, cultural activities in	Students more able to apply new knowledge of Indigenous oral health and Maori health in practice

			Maori tribe curriculum development partnership	local Maori community	
Carter & Rukholm (2009) [50]	Evaluation; Semi-structured interviews; qualitative analysis of online discussion forum; Interdisciplinary Education Perception Scale (pre-and post)	Canada; n=6 Nursing, Medical, Health Promotion, Social Work	Collaboration with four universities; Aboriginal community Elders directed curriculum	Online interprofessional learning module. Video clips of Elders speaking; glossary, case-based cultural learning activity; Guided Listening Tool; bulletin board/ discussion forum	All students reported increased awareness of Aboriginal people's health beliefs/attitudes, and interprofessionalism; increased personal growth
Chiodo and colleagues (2014) [15]	Open-ended evaluation questionnaire	Australia; n=113 second-year psychology class	University tutors voiced necessity of cultural unit	6-week unit; weekly 2-hour lectures; weekly 1-hour tutorial; Indigenous guest speakers; articles led by Indigenous authors; use of case studies and digital resources; reflective journaling	Increased awareness on cultural diversity issues; greater understanding of past and present racism; increased self-awareness, white privilege. Some recognition unit content helpful in becoming culturally competent practitioners; some too much focus on Indigenous issues (student resistance)
Cross and colleagues (2015) [39]	Program evaluations of 6 Child Welfare Tribal Traineeship Programs	United States; 5 universities; 1 college; bachelor or masters of social work degrees; n=22 tribal students; n=58 non-tribal students	Offered by the National Child Welfare Workforce Institute; all tribal traineeship programs partner with state agencies, tribal nations, tribal agencies, faculties and Social Work professionals	Designated child welfare field placements; engagement in community events, co-training with child welfare professionals, attendance and co-presentations at national conferences; sharing circles	Development of cultural sensitivity in American Indian child welfare knowledge; recognition of importance of mentorship and cultural responsiveness; non-tribal students became workforce allies
Dowell and colleagues (2001) [55]	Student questionnaire within standard university course evaluation	New Zealand; n=51 3rd year Medical	Student experience coordinated by local Maori health provider; Elder assisted cultural proceedings	One week immersion in six small Maori communities; needs assessments; presentation assignment for class and community representatives	Community health needs assessment and cultural immersion highly effective in student learning especially when taught by Maori people; students more interested career options in communities
Duthie and colleagues (2013) [20]	Socio-cultural constructivism; Informal conversation and open-ended questionnaire	Australia; Masters of social work	Indigenous unit coordinator ensured community members would benefit from students' visits; consultation with an Elder	1-day field experiences to Indigenous Community Northwest of Brisbane; communication with community members and agency visits	Increased self-awareness and socialization skills with Indigenous people; increased understanding of colonization and its' impacts; stereotypes challenged through real life experience and conversation
Hart and colleagues (2015) [21]	Mixed methods; quantitative and qualitative online survey; Thematic analysis	Australia; nursing; n=17 Placement completions; n=8 Survey completions	Consultation with Indigenous Medical Services; university partnership with primary healthcare workers and Indigenous communities	Strengthening Nursing Culture - Indigenous Medical Services Placements project; 5-week clinical placement	High levels of student confidence as a result of Indigenous community placement; increased cultural humility and competency; support from community/placement and university staff important

Hays (2002) [22]	Description	Australia; Medical	Local Indigenous community members on committees to design curriculum, select students/staff	Employed Indigenous staff role models; Indigenous content over 6 years; seminars, tutorials, community placements	Partnership approach strategies and recommendations; no findings reported
Hendrick and colleagues (2014) [23]	Critical theory and empowerment framework; Observation	Australia; first-year interprofessional - Social Work, Nursing, Midwifery, Physiotherapy, Medical Imaging, Psychology, Health and Safety, Occupational Therapy and 20 other disciplines; n=2000 (approx.) per unit	Partnership with the Center for Indigenous Studies and Indigenous staff; input from Indigenous communities	Indigenous cultures and health (ICH) unit; 12 weekly 2-hour workshops; critical engagement and reflective journaling; unit coordination by 1 Indigenous person and 1 non-Indigenous person; Indigenous voices present in unit; tutor support by Indigenous and non-Indigenous people	Unit shines light on 'educational blind spot' i.e. Stolen Generations; critical reflection challenges interests of dominant groups; recognition of importance of ICH unit; process of becoming a critical reflective practitioner
Hudson and Maar (2014) [51]	Pilot study; reports and post-pilot symposium	Canada; n=15 Medical and nursing students	Collaboration with Northern Ontario's Aboriginal political bodies and organizations	Pilot mandatory placements in Aboriginal communities; 2-week placement + 2 weeks on campus with web-based interaction	Students require more in-class preparation before immersion; lack of meaningful discussion online; stereotypes challenged in placement; cultural learning; recognition of community health and advocacy needs; pilot study results led to future mandatory placements in Aboriginal communities
Hunt and colleagues (2015) [24]	Mixed-methods	Australia; n=944 Nursing, n=502 completed baseline survey; n=249 completed follow-up survey	Not discussed	Course unit related to history, culture and health of Australian Indigenous people	Decrease in negative attitude scores; increase scores on knowledge, interest and confidence working with Australian Indigenous people; enhanced respect and cultural competence; sample may not be representative given low response rates
Isaacs and colleagues (2016) [25]	cross-sectional comparison of cultural competence and skill; learning of Indigenous content and cultural desire	Australia; n=220 2-year Nursing; n=109 in unit; n=111 not in unit; Survey	Not discussed	10-week Indigenous health and well-being unit; and 9-hour long weekly lectures and tutorials for discussion	Overall opinion that unit is necessary regardless of prior unit completion; Students who completed unit had increased understanding of Indigenous Health; cultural desire difficult to measure
Isaacson (2014) [40]	Mixed-methods; Hermeneutic Phenomenology; descriptive and inferential statistics	United States; 4-year Nursing; n=8 Group 1; n=3 Group 2	Not discussed	American Indian Northern Plains Reservation immersion; Group 1, 4-day Health screenings at Public School; Group 2, 2-week 80-hour clinical practicum at Indian Health Service Facility; Reflective journaling	Following immersion, students reported they were not as cultural competent as they assumed prior to practicum; increased awareness of power imbalances in healthcare

Jackson and colleagues (2013) [26]	Evaluation; survey	Australia; n=56 post-graduate students from Health disciplines; primarily nurses and midwives	Collaboration between a single non-Indigenous and four Indigenous academic nurses	1-day workshop on health & social disparities and positive & affirming images of strength and resilience; Transformative learning; Critical-dialectical discourse and group work; filmed documentary	Seen as transformative, profound, deeply meaningful and useful and relevant to practice; Day was emotionally draining
Jarvis-Selinger and colleagues (2008) [52]	Description	Canada; Medical, Pharmacy, Nursing, Social Work	Social accountability model collaboration with community health administrators, Aboriginal community members, health professionals, policy makers; Aboriginal leaders on Steering Committee	Interprofessional 4 week community immersion taught by Aboriginal university and community instructors; reading assignments, discussion groups, reflective journal, community health priority presentations of individual and team projects	Identified need to integrate interprofessional health curricula (Aboriginal perspectives, social accountability); administrative and curricular support, interprofessional scheduling, cross-discipline understanding and communication highlighted
Joyce (1996) [56]	Programme Evaluation	New Zealand; Polytechnic based Nursing program	Based on Wood and Schwass' (1993) model	Cultural safety 7% of programme hours. Small groups co-taught by lecturers, one Maori teacher	Students gain greater acceptance of different world views in first year; students attitudes more accurate in practice areas rather than classrooms. Educators need to have significant knowledge and skills to deal with student attitudinal issues.
Kickett and colleagues (2014) [27]	Descriptive; quantitative and qualitative student feedback	Australia; mandatory first-year unit in Health Sciences faculty; n=2300 (approx.) annually; n=1873 in 2011	Co-ordination of unit by an Indigenous staff member and a non- Indigenous staff member; Indigenous tutors available to students	Online or face-to-face course; 2-hour tutorials over 12 weeks; podcasts featuring Indigenous people; group presentations and class discussions, e-tests; reflective journaling	Course provided new content and a perspective that many students had not been previously exposed to; some white students felt persecuted
Kline and colleagues (2013) [53]	Semi-structured interviews	Canada; n=54; between 2006 & 2009; Medicine, Nursing, Occupational Therapy, Social Work, Pharmacy, pre-Medicine, Midwifery, Dietetics, Dentistry, Land and Food Systems, Psychology	University request for Aboriginal community members and program supervisor of Aboriginal Child & Family Services Agency of the Stó:lō Nation	Summer camps for Aboriginal youth; led by elders, youth workers and cultural leaders; camp held on reserve in longhouse or outdoors; students participated in activities, organizing and providing basic first aid; facilitated discussion on health topics with discretion of camp leaders and youth	Developed self-awareness, cultural humility and respect; greater understanding of colonial history, time and trust; students and community members were able to recognize the health expertise that exist in the community
Lalloo and colleagues (2013)	Evaluation; Reflective journals; online survey	Australia; n=156 Dentistry – final year	Not discussed	Rural Indigenous clinical immersion	Students report positive learning experience. Application of knowledge/learning will be

[28]		of preregistration 2009 to 2011			realized in rural clinical practice after graduation
Mak and colleagues (2006) [29]	Evaluation; Reflective journals; Interviews; Follow-up questionnaire 6 months to 2 years after completion	Australia; Undergraduate prevocational Medical practitioners (PMPs)	Not discussed	4-week metropolitan sexual health clinic and 20-week remote public health/ primary care settings. Curriculum based on sexual and rural health in Indigenous setting;	PMPs developed awareness of determinants of health in relation cultural setting, health system, and complexity of health promotion healthcare delivery in remote areas and Indigenous settings;<br>PMPs reported professional and personal growth
Morrissey & Ball (2014) [30]	Questionnaire analysis and analysis of student narratives	Australia; n=29; Pharmacy and Clinical Sciences	Not discussed	Cultural awareness training then visits to a number of Health Facilities and Indigenous communities; group discussion; reflective journaling; evening workshops	Modern Racism Scale (MRS) showed that students empathy towards Indigenous Australians improved post-visits; the Attitudes Towards Indigenous Australians Scale (ATSI) also improved
Nash and colleagues (2006) [31]	Description; Evaluation Pre-evaluations, survey questionnaires, focus groups used to develop curriculum; Pre/Post faculty assessment using Indigenous Perspectives Tertiary Educator's Awareness, Knowledge, and Skills Scale	Australia; N=89 undergraduate Nursing and Faculty	Consultation and collaboration with Indigenous staff/local Indigenous and Torres Strait Islander, community health experts. External health stakeholders, Indigenous clinicians, nurses teach content/assessment strategies	Concepts embedded into existing courses; website with teaching/learning services. Intensive professional development workshops for teaching staff	Faculty initially tentative about course content gained confidence from involvement of Indigenous consultants and workshops. Cultural competence should be ongoing - not mastered in one course, semester, year
Paul (2006) [32]	Evaluation; questionnaire on self-perceptions of preparedness and future commitment in working in Indigenous health.	Australia; n=125 final year Medical students; 2 cohorts (2003 and 2004)	Not discussed	Integrated Indigenous health curriculum throughout 6 year program; seminars, guest lectures, problem-based tutorials, self-directed, and practice placement	Significant student self-perceived levels of knowledge, skills and attitudes about Aboriginal health/culture with small amount of targeted and structured teaching and learning in Indigenous health. suggest rural immersion not necessary
Pickrell (2001) [41]	Description; no formal evaluation component; some faculty observations and verbal feedback reported	United States; Psychology, OT and 3rd year Nursing	Not discussed	Classroom instruction; 15 hours; American Indian Lakota cultural values and practices; minimum 45 clinical hours on reservation	Students examined own beliefs/values worldview; immersion in Lakota culture increased ability to provide culturally sensitive care
Prout and colleagues (2014) [33]	Experiential Education Theory, Situated Learning, and Transformative Education	Australia; n=27 Nursing, Physiotherapy, Health Promotion, Health Science, Pharmacy, Social Work, Medical Imaging	Coordinated by University's Center for Rural health	"Country week"; 1-week facilitated intensive immersion; attended community audits, interactive meetings with community teachers and a	Increased self-awareness and transformative learning; recognition of immersion experience as deeper learning than in the classroom

				pilgrimage through places of historical significance; reflection journaling	
Ranzijn and colleagues (2008) [34]	Evaluation: Standard course evaluation questionnaire; (40% response rate - similar to university wide response rate)	Australia, n=220 Year 1 undergraduate Psychology	Reference interdisciplinary group including Indigenous people involved	Compulsory 13-week Indigenous Studies course; weekly 2-hour lecture/1-hour tutorial (colonization, cultural competence, transgenerational trauma and psychological impacts) taught by Indigenous/non-Indigenous lecturers/tutors	Indigenous teachers sharing of personal experiences critical for learning. Balance of Indigenous/non-Indigenous teaching appropriate and valuable. Students who would not have taken course if optional, pleased they did. Integration of content important for Indigenous-related cultural competence. Institutional faculty resistance to Indigenous content
Roche and colleagues (2007) [42]	Evaluation; Electronic survey	United States; n= 15 Pharmacy	Not discussed	16 week elective course; 2 hrs per week; Readings, reflective journals, discussions with Native American/non-Native American guest speakers; Oral presentations on Native American health issues; cultural immersion practicum experience	Lasting impact on students' understanding of Native American social/healthcare issues, their practice and personal lives. Most students sought additional learning experiences with tribal people and employment in Indian Health Services following the course
Roche (2014) [43]	Long-term Evaluation	United States; n=20 Pharmacy	Not discussed	Pharmacy unit analyzing Native American healthcare beliefs, traditions, and disparities; readings and interactions with healthcare practitioners and Healers; reflective journaling; talking circles; fall break spent in Navajo nation with 2 days at a healthcare facility	11-year review of pharmacy unit; many students graduated and began working in Indian Health Services; increased awareness of social and health challenges and its' impacts
Smith and colleagues (2015) [35]	One-year evaluation feedback	Australia; In 2012, n=92 Medical students	High-level multicultural and multidisciplinary Indigenous health group; 2 Indigenous doctors, 1 Indigenous Elder and educator, and 1 doctor who worked in an Indigenous medical service partnered with 5 academic staff (2 educationalists, a clinical ethicist, an anthropologist, and a research psychologist)	Prerequisite lectures, then a 1.5 day cultural immersion; cultural education sessions; storytelling and culture and identity sessions; cultural evening festival	Positive feedback contributed to university's continuation of cultural immersion; greatest strength of immersion was the development team; students felt more confident to communicate with Indigenous, including Torres Strait Islander peoples and identify connection between history and health outcomes

Thackrah and colleagues (2014) [36]	Evaluation; thematic analysis of in depth, semi-structured, face-to-face interviews	Australia; n=7 Midwifery	Not discussed	Up to 2-week clinical placement to the Aboriginal Ngaanytjarra Lands community	Students valued connections made with Indigenous women and children; recognition of lack of medical service access in rural location; respect for traditional knowledge around birthing and family practice
Walton (2011) [44]	Evaluation; pre-/ post-surveys; qualitative critical reflection paper	United States; n=65 Health Science; n=30 Nursing	Not discussed. Involvement of Native Americans not evident	One hour case based study presentation about a young Native American living with chronic renal disease; peer-review reading;	Students learn cultural awareness and application of culturally aware interventions
Warner (2002) [45]	Description; daily post-clinical conference reflections	United States; n=10 Nursing	Curriculum developed in partnership with community Public Health Native American Navajo Nurses and workers	Part of full clinical course; nurse preceptorship and public health Navajo worker shadowing. Cultural immersion living in a remote rural Navajo community; faculty facilitation role; Seminars of team building and cultural learning specific to the immersion experience and public health	Students reported personal growth, better understanding of provider/patient power differentials, and culture within non-Western healthcare perspective. Faculty need to review own attitudes and abilities; supportive academic environment necessary for student learning
Warren (2003) [57]	Evaluation; phenomenological approach (Van Manen, 1990); semi–structured interviews	New Zealand; n=10 3rd year Nursing (6 women/4 men; 1 Māori)	Not discussed.	Delivery not discussed but teaching concepts based on safety curriculum adopted by Nursing Council of New Zealand	Cultural safety concept not well articulated by students; improved understanding of attitude and impact of unsafe cultural practices. Increased knowledge of own culture and impact on practice. Students more willing to change attitude. More accepting of client's choice of Māori health practices
Wendler & Struthers (2002) [46]	Evaluation; Shortened version of standard class midterm and final evaluation; reflective faculty and course evaluation	United States; Nursing; n=51 undergraduate; n=7 graduate	University wide requirement of cultural diversity content. Caucasian, middle-class nursing faculty with knowledge and skill of working with diverse culture developed original cross-cultural health course; doctoral prepared Native American nurse coach and online dialogue as non-evaluative faculty member	Web-based delivery. Journal; culturally specific and relevant readings; discussion groups; engagement in cultural enrichment activity, course paper or project with an executive summary	Students rated course very positively almost all of the course evaluation prompts. Students appreciated cultural-enrichment activity and synthesis of course learning into paper or project
Wittig (2004) [47]	Evaluation: based on Campinha-Bacote (2002) cultural	United States; Nursing; Final semester (n=28; 1 male, 27 females)	Not discussed	Classroom content included knowledge of the basic beliefs of some groups (i.e., Native Americans); guest	Students better able to understand culture and cultural health practices, appreciation of all groups; spiritual and religious beliefs; health and risk

	competence model (assessment of perceptions, beliefs, practices and culturally competent nursing care for Native American clients)	11 (aged 18 to 25; 9 (aged 26 to 35); 8 (over 34 yrs); 71% provided care to Native American clients		speakers from a tribe involved in teaching	factors, and self-knowledge; non-judgmental caring attitude; respect for diversity. Native teachers in class important
